# Beyond Wayfinding: The Role of Navigation Technology in Supporting Emotional Well-Being

**DOI:** 10.1093/geroni/igaf122.1999

**Published:** 2025-12-31

**Authors:** Bisola Adeleke, Shannon Mejia

**Affiliations:** University of Illinois Urbana-Champaign, Champaign, Illinois, United States; University of Illinois Urbana-Champaign, Champaign, Illinois, United States

## Abstract

The ability to navigate successfully is essential for maintaining independence, autonomy, and social engagement, which are fundamental to healthy aging. However, age-related change in cognitive and physical function can reduce confidence and limit older adults’ willingness to commute, thereby affecting their quality of life. While modern navigation technologies offer potential solutions to support independence, the role in enhancing emotional well-being in older adults remains underexplored. This study, grounded in the Technology Acceptance Model (TAM), explored the implications of navigation use for perceived constraints, travel, and affect among older adults. Using data from the 2022 Health and Retirement Study (N = 4,074), we applied structural equation modeling (SEM) to test reduction in perceived constraints on personal control and travel/commuting yesterday as serial mediators of the association between frequency of navigation use and yesterday’s positive affect, with age, financial strain, and self-reported ongoing health problems as covariates. Findings revealed that more frequent navigation technology use was directly associated with greater positive affect. A significant indirect effect (β = 0.064, p = 0.001) indicated that navigation technology use also supported positive affect through lower perceived constraints, which increased the likelihood of travel/commuting yesterday, and in turn by increasing the likelihood of travel through lower perceived constraints on personal control. Taken together, our findings suggest that digital navigation tools play a role beyond wayfinding, and also support well-being through psychological pathways. Attention to preservation of autonomy may play a critical role in enhancing perceived usefulness and support sustained adoption and use of navigation technologies.

